# Natural Product-Based Studies for the Management of Castration-Resistant Prostate Cancer: Computational to Clinical Studies

**DOI:** 10.3389/fphar.2021.732266

**Published:** 2021-10-19

**Authors:** Rajeev K. Singla, Pooja Sharma, Ankit Kumar Dubey, Rohit Gundamaraju, Dinesh Kumar, Suresh Kumar, Reecha Madaan, Richa Shri, Christos Tsagkaris, Salvatore Parisi, Shikha Joon, Shailja Singla, Mohammad Amjad Kamal, Bairong Shen

**Affiliations:** ^1^ Institutes for Systems Genetics, Frontiers Science Center for Disease-Related Molecular Network, West China Hospital, Sichuan University, Chengdu, China; ^2^ iGlobal Research and Publishing Foundation, New Delhi, India; ^3^ Department of Pharmaceutical Sciences and Drug Research, Punjabi University, Patiala, India; ^4^ Khalsa College of Pharmacy, Amritsar, India; ^5^ Institute of Scholars, Chikmagalur, India; ^6^ ER Stress and Mucosal Immunology Lab, School of Health Sciences, College of Health and Medicine, University of Tasmania, Launceston, TAS, Australia; ^7^ Department of Pharmaceutical Sciences, Sri Sai College of Pharmacy, Amritsar, India; ^8^ Chitkara College of Pharmacy, Chitkara University, Punjab, India; ^9^ Faculty of Medicine, University of Crete, Heraklion, Greece; ^10^ Lourdes Matha Institute of Hotel Management and Catering Technology, Thiruvananthapuram, India; ^11^ West China School of Nursing/Institutes for Systems Genetics, Frontiers Science Center for Disease-Related Molecular Network, West China Hospital, Sichuan University, Chengdu, China; ^12^ King Fahd Medical Research Center, King Abdulaziz University, Jeddah, Saudi Arabia; ^13^ Enzymoics; Novel Global Community Educational Foundation, Hebersham, NSW, Australia

**Keywords:** castration-resistant prostate cancer (CRPC), hormone-sensitive cancer, advance staged cancer, natural products (NP), natural anticancer agents, tumor microenvironment, cheminformatics

## Abstract

**Background:** With prostate cancer being the fifth-greatest cause of cancer mortality in 2020, there is a dire need to expand the available treatment options. Castration-resistant prostate cancer (CRPC) progresses despite androgen depletion therapy. The mechanisms of resistance are yet to be fully discovered. However, it is hypothesized that androgens depletion enables androgen-independent cells to proliferate and recolonize the tumor.

**Objectives:** Natural bioactive compounds from edible plants and herbal remedies might potentially address this need. This review compiles the available cheminformatics-based studies and the translational studies regarding the use of natural products to manage CRPC.

**Methods:** PubMed and Google Scholar searches for preclinical studies were performed, while ClinicalTrials.gov and PubMed were searched for clinical updates. Studies that were not in English and not available as full text were excluded. The period of literature covered was from 1985 to the present.

**Results and Conclusion:** Our analysis suggested that natural compounds exert beneficial effects due to their broad-spectrum molecular disease-associated targets. *In vitro* and *in vivo* studies revealed several bioactive compounds, including rutaecarpine, berberine, curcumin, other flavonoids, pentacyclic triterpenoids, and steroid-based phytochemicals. Molecular modeling tools, including machine and deep learning, have made the analysis more comprehensive. Preclinical and clinical studies on resveratrol, soy isoflavone, lycopene, quercetin, and gossypol have further validated the translational potential of the natural products in the management of prostate cancer.

## Introduction

With the global burden of cancer increasing at an alarming rate, health systems struggle to find any cost-effective strategies, particularly in poor and developing countries (Suryanarayana et al., 2015). Despite advancements in screening and early diagnosis strategies, about 20% of men have prostate cancer (PCa). PCa is predominantly controlled by androgen-binding and transcription signals from the androgen receptor (AR) (Studer et al., 2004; Singer et al., 2008; Padmanabha and Hariharan, 2016). Men with PCa usually respond to androgen deprivation therapy (ADT). However, in most individuals, the illness progresses remarkably within 2 years, which has been termed castration-resistant prostate cancer (CRPC). CRPC is a kind of advanced PCa that progresses with the circulating testosterone levels (<50 ng/dl) after being castrated surgically or pharmaceutically (Hotte and Saad, 2010; Kirby et al., 2011). Most individuals with advanced disease acquire resistance to ADT and develop CRPC (Sarkar et al., 2010). Although it has not been fully understood how the prostate cells become castrate-resistant, one of the accepted reasons is the deprivation of androgen that eventually gives androgen-independent cells a selection advantage, thus allowing them to flourish and eventually recolonize the tumor (Hoimes and Kelly, 2009). However, studies have reported that PCa cells could overcome castration-induced growth inhibition by upregulating enzymes that promote androgen production in tumor tissue (Grossmann et al., 2001). The realization that CRPC still plays a significant function in the androgen axis has prompted more research and development of therapy methods (Kirby et al., 2011; Chandrasekar et al., 2015). Even though new therapy options for individuals with advanced-stage PCa have recently been authenticated, the fact remains that CRPC is catastrophic (Lian et al., 2015).

Recent research developments have resulted in significant progress in strategies for managing PCa in terms of diagnosis and treatment. Complementary and alternative medicines (CAMs), including those from plant, animal, and microbial sources, are eliciting strong potential for the treatment management of various diseases and disorders, including cancer (Al-Menhali et al., 2015; El Hasasna et al., 2015; Athamneh et al., 2017; Fardoun et al., 2017; Elmas et al., 2018; Song et al., 2019; Nishimura et al., 2021). Pieces of evidence have supported the utility potential of CAMs; therefore, clinical studies were carried out so that CAMs would reach the patient’s bedside. With advances in organic chemistry and chemical analysis, the analytical investigation has opened the door to the isolation/purification and characterization of numerous active compounds of plants (Newman et al., 2000; Singla et al., 2020a; Shen and Singla, 2020). One significant advantage of medicinal plant-based drug development is the availability of ethnopharmacological data, which can be used to narrow down the vast number of probable leads and choose the most promising ones (Choudhari et al., 2020; Singla, 2020). However, the integrated drug discovery approach supported by multidisciplinary fields, including medicinal chemistry, pharmacology, natural product chemistry, biochemistry, and molecular and cellular biology, is expected to lead to a better understanding of the potential of phytochemicals (Newman and Cragg, 2016; Singla et al., 2020b; Choudhari et al., 2020; Singla and Shen, 2020). A large number of phytochemicals, such as quercetin (Rauf et al., 2018), fisetin (Lall et al., 2016), curcumin (Ide et al., 2018), genistein (Basak et al., 2008), resveratrol (Lee et al., 2014), have been found to modulate AR activity and expression. Bioactive metabolites produced from edible plants (nutraceuticals) and traditional folk sources are potentially multitarget and are thus preferred over the primarily single-target anticancer agents, such as kinase inhibitors (Kallifatidis et al., 2016).

## Castration-Resistant Prostate Cancer

### Epidemiology

**FIGURE 1:**
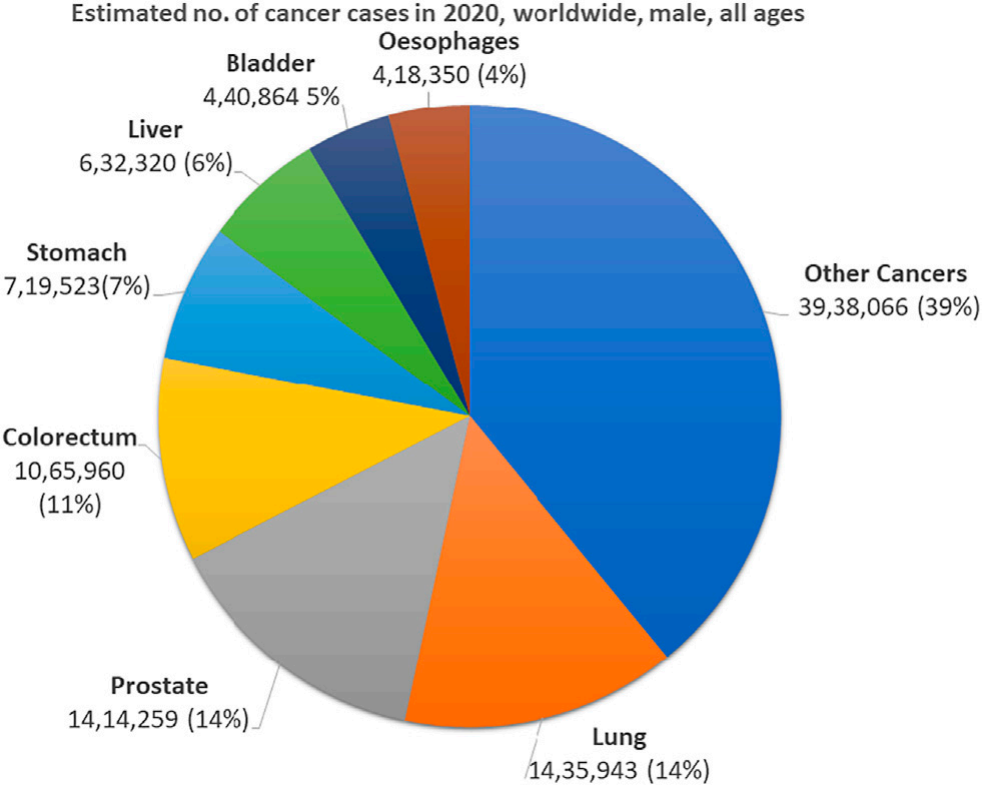
Pie chart depicting the distribution of global prevalence of the seven most frequent cancers in 2020 for all ages. Adapted from the data published by Wild et al. (2020).

PCa is the second most common cancer in males and the fifth-greatest cause of cancer mortality in 2020, with an anticipated 1.4 million new cases and 375,000 deaths globally (Figure 1) (Lin et al., 2018; Sung et al., 2021). It is considered the second leading cause of cancer-related male fatalities and the most common noncutaneous malignancy in males, with more than 160,000 new cases reported in 2017 in the United States (Zhu et al., 2015; Howlader et al., 2017; Teo et al., 2019). According to the GLOBOCAN database, in 2018, an estimated 1.2 million new cases of PCa were recorded worldwide, with developed nations having a greater prevalence. Moreover, the global burden is expected to increase to over 2.3 million and 7,40,000 deaths by 2040 (Ferlay et al., 2018; Rawla, 2019; Culp et al., 2020). PCa is prevalent in almost all major countries and is the leading cancer-related cause of death in males in over 100 nations after lung cancer (112 of 185). The incidence rate ranges from 6.3 to 110.4 per 100,000 males across countries. The higher rates were observed in Northern and Western Europe, the Caribbean, Australia/New Zealand, Northern America, and Southern Africa, whereas the lowest rates were found in Asia and Northern Africa. Previous research studies have suggested that African-American males have the highest prevalence of PCa worldwide and are more prone than other racial and ethnic groups to get the disease at a younger age (Kheirandish and Chinegwundoh, 2011). The higher rates may indicate a greater disease incidence and higher rates of PCa than other places across the globe (Wild et al., 2020; Sung et al., 2021). For a prevalent illness like PCa, little is known about its genesis, and only a few risk factors have been discovered (Lin et al., 2021). Several factors are responsible for changes in its prevalence at the regional level due to changes in the susceptibility of different population groups to environmental risk factors, including racial/ethnic backgrounds, geographical heterogeneity, advancing age and an intact hypothalamic-pituitary-gonadal axis, family history, genetic mutations (e.g., BRCA1 and BRCA2), and diagnosis and access to good quality treatment (Hoimes and Kelly, 2009; Rebbeck et al., 2013; Shackleton et al., 2021). PCa incidence and death rates differ significantly between ethnic groups, implying ethnic and genetic susceptibility (Shackleton et al., 2021). However, since the 1990s, mortality rates in PCa have declined in most high-income countries, including Northern America, Oceania, and Northern and Western Europe, due to advances in therapeutics and earlier diagnostics using enhanced screening methods (Tsodikov et al., 2017). This diversity in PCa mortality rates throughout the world is partly due to underlying biological disparities in risk and treatment availability. For example, places with higher diagnosis rates of low-grade malignancies and improved treatment choices (such as Northern America and Asia) have lower death rates than those with poor screening rates, concomitant diagnoses of aggressive tumors, and limited treatment choices (such as Sub-Saharan Africa).

### Tumor Microenvironment in Castration-Resistant Prostate Cancer

Immune cells are key components of the tumor microenvironment associated with tumor progression. T regulatory cells (Tregs), tumor-associated macrophages (TAMs), tumor-infiltrating B lymphocytes (TILs), neutrophils, and myeloid-derived suppressor cells (MDSCs) are part of the infiltrated immune cell of the prostate tumor microenvironment. However, various studies have reported that the progression of PCa is influenced by the tumor-associated immune cells and inflammatory cytokines, such as IL-23 (Ammirante et al., 2010; Si et al., 2013; Calcinotto et al., 2018; Wang et al., 2019; Zhang Z. et al., 2020). TAMs are a major component in the development of PCa, though the specific pathway for the release of cytokines, matrix metalloproteinases, and growth factors is still unknown (Shimura et al., 2000; Arora et al., 2018). Castration resistance is characterized by both hyper- and/or constitutively active androgen receptor expression (AR) in PCa cells toward cellular interactions between stem cells and bone microenvironmental systems (Karamanolakis et al., 2016). The cancer cell dependency on the tumor microenvironment indicates that the noncancer cell component of the tumor can regulate the spread of PCa. However, the immune response from the tumor microenvironment contributing to the development of CRPC is unknown (Calcinotto et al., 2018). The typical prostate gland comprises prostatic ducts surrounded by epithelium and a stroma made up of smooth muscle cells with a few fibroblasts, endothelial cells, and nerve cells. ARs are widely expressed in several normal prostate stromal cell types, including smooth muscle cells, endothelial cells, and epithelial cells (Cunha et al., 1996). Multiple nonmalignant cells, such as fibroblasts, myofibroblasts, endothelial cells, and immune cells, chemokines, cytokines, growth factors, extracellular matrices (ECMs), and matrix-degrading enzymes make up the stromal compartment (Corn, 2012). The connection between the epithelial and stromal sections facilitates the progression of tumors through processes such as ECM reintegration, increasing penetration and releasing soluble growth factors for castrate-resistant growth, and angiogenesis stimulation (Rowley, 1998; Karlou et al., 2010). Immune cells are typical inhabitants and have a protective function against infections that infiltrate healthy prostatic tissue. However, histological investigations have revealed that high-grade PCa is associated with enhanced stromal immune cell infiltration with differences between tumor-stage cell types (Gurel et al., 2014).

### Signaling Pathways Orchestrating Castration-Resistant Prostate Cancer

One of the factors contributing to PCa progression is androgen binding to androgen receptors. Herein, androgens play a pivotal role in the growth and survival of PCas. Moreover, increased expression of androgen receptors is often observed in CRPC (Gelmann, 2002). Androgen receptor translocates into the nucleus with the aid of ligand binding, where it orchestrates transcription, modulates growth signaling pathways, and dictates programmed cell death, cellular proliferation, and androgen-associated genes (Andersen et al., 2010). PI3K-Akt-mTOR pathway stands at the forefront of the initiation of CRPC, which is essentially triggered by G protein-coupled receptors (GPCR). Accumulating evidence has suggested that perturbations in the PI3K-Akt-mTOR pathway occur in the vast majority of metastatic cancers; hence, PI3K-Akt-mTOR can be a potential therapeutic target (Huang et al., 2018). Phosphatase and tensin homolog (PTEN) has been associated with PI3K-Akt-mTOR triggering in advanced stages of the disease, especially the downregulation of this gene (McMenamin et al., 1999). A previous study has proven that AR is interlinked with PI3K-Akt-mTOR, where combination therapy of AR and PI3K-Akt-mTOR inhibition by EPI-002 and BEZ235 *in vitro* and *in vivo* successfully reduced LNCaP95 cell growth and was noted as a potential therapeutic avenue in CRPC (Kato et al., 2016).

Similarly, the JAK/STAT pathway is also a membrane-to-nucleus signaling pathway, which is vital for the development, proliferation, migration, and apoptosis of cells, often triggered by agents, such as cytokines and growth factors (Ramalingam et al., 2017). When triggered, JAKs induce phosphorylation of STAT proteins. Therefore, STATs are dimerized and thereby translocated to the nucleus *via* importin α-5 and the Ran nuclear import pathway. STATs display a specific stimulation or suppression of transcription of target genes by binding to specific sequences inside the nucleus (Ramalingam et al., 2017). JAK/STAT3 pathway is a well-established prosurvival inducing mechanism, where repression of this pathway reduced the PCa cell growth and induced programmed cell death (Liu et al., 2012). Oncogenes like BRAC induce proliferation and migration *via* the JAK/STAT3 pathway (Gao et al., 2001). Furthermore, STAT3 activation triggers other genes linked to the cell cycle, angiogenesis, and tumor progression (Dhir et al., 2002; Zhu and Kyprianou, 2008). In various conditions, JAK/STAT3 is a predictor of poor disease prognosis (Liu et al., 2012).

Src signaling has been implicated in promoting growth factors and inflammatory cytokines, such as IL-8, and inducing angiogenesis (Park et al., 2007). Additionally, Src signaling triggers nuclear factor-KB (Nf-KB) and tumor necrosis factors involved in evading apoptosis and promoting bone metastasis of PCa (Park et al., 2007). Targeting Src has immensely slowed down tumor growth and hindered invasion, which can be achieved using the two clinical drugs, dasatinib and saracatinib (Yang et al., 2010; Araujo et al., 2013). Moreover, growth factors, such as IGF-1, IL-6, and EGFR, can individually promote CRPC (Bettedi and Foukas, 2017). Activation of Her-2/neu, a receptor tyrosine kinase, has been observed to increase the growth of CRPC in clinical samples and xenograft models (Wen et al., 2000; Neto et al., 2010). A clinical study on targeting growth factor signaling pathways with the help of cabozantinib and tyrosine kinase inhibitor has achieved a therapeutic advantage in various stages, such as survival devoid of disease progression and marked decrease of metastatic lesions (Smith et al., 2013).

Recent evidence has revealed a regulatory mechanism, that is, crosstalk between AR and Wnt pathway, where suppression of Wnt pathway facilitates reducing AIPC cell growth by inhibiting cell cycle progression and promoting apoptosis *in vitro*. Further, a correlation between Wnt genes, like WNT5A and LEF1, and metastatic PCa has been observed (Luo et al., 2020). Wnt catenin pathway plays a principal role in homeostasis, proliferation, migration, and cell transitions (Kuhl and Kuhl, 2013). Increased catenin in PCas leads to phosphorylation and inactivation of GSK3 (Chesire and Isaacs, 2003; He et al., 2004). Inclined expression of Wnt is correlated with disease progression and metastasis. Furthermore, FZD4 overexpression favors EMT in CRPC (Polakis, 2007; Gupta et al., 2010).

**FIGURE 2:**
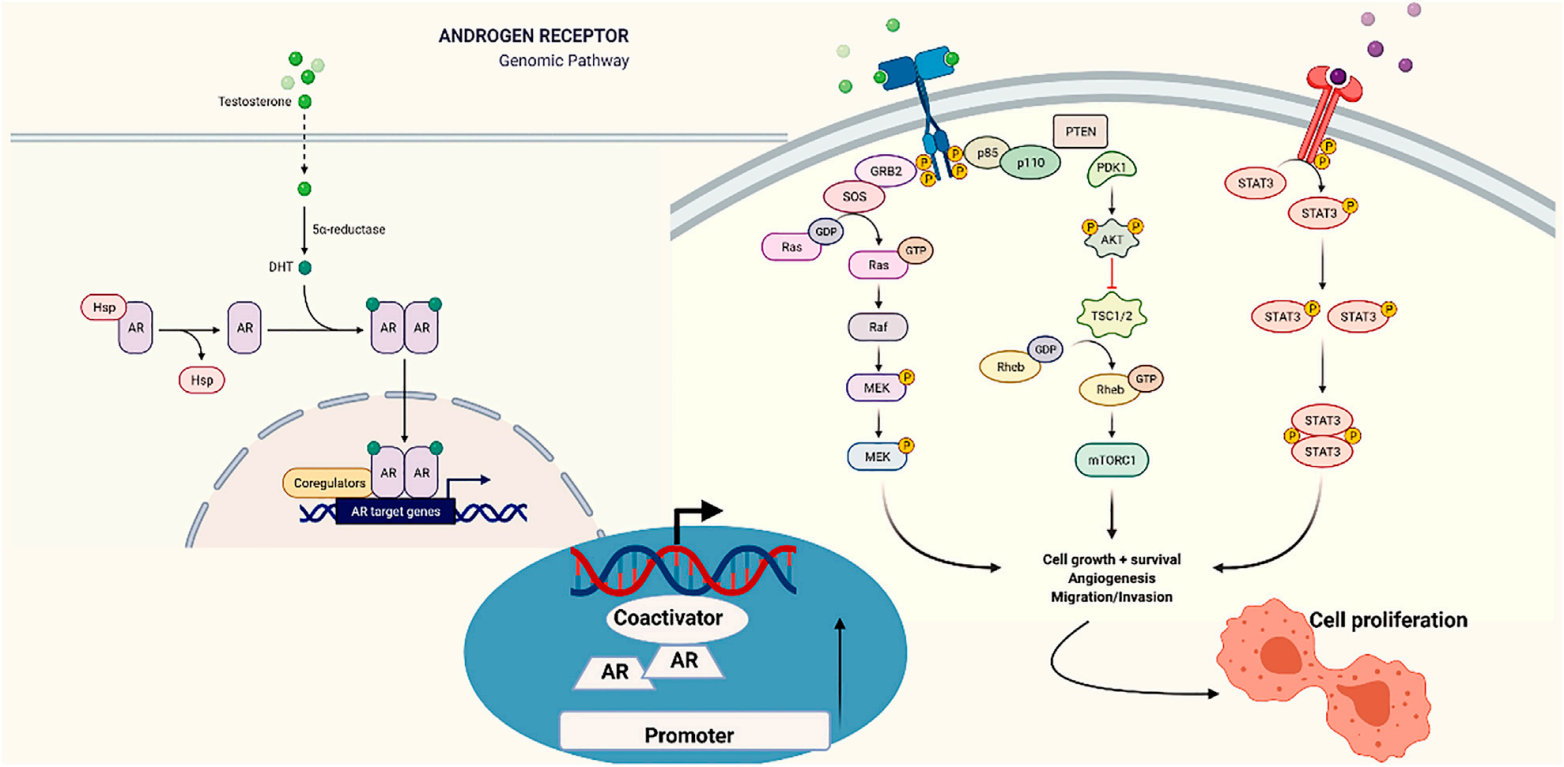
Signaling pathways in castration-resistant prostate cancer.

Long noncoding RNAs (lncRNAs) are a group of transcripts widely employed as diagnostic tools and have received great attention because they are expressed in a more tissue-specific manner. Recent emerging evidence has shown that lncRNAs play vital roles in cancer initiation and progression, including PC progression and AR-related pathways (Ren et al., 2013; Fang et al., 2016). In a very recent study by Yao et al., LINC00675 has been abundantly found in both androgen-insensitive cells and CRPC patients. In the same study, it has been confirmed that LINC00675 binds to GATA2 mRNA and makes GATA2 an activator in the AR signaling mechanism in the nucleus, contributing to both castration resistance and disease progression (Yao et al., 2020). Signaling pathways associated with castration-resistant prostate cancer are illustrated in Figure 2.

An androgen receptor-dependent mechanism of resistance in hormone-naïve PCa leads to castration resistance. Apart from the AR, survival can be achieved and enhanced via cell-intrinsic pathways or progrowth signals from the microenvironment.

### Genomic Targets of Phytomolecules Against Castration-Resistant Prostate Cancer

Expansion of malignant prostatic cells and normal cells and proliferation of CRPC are extremely dependent on androgens. Consequently, it has been hypothesized that androgens play a fundamental role in prostate tumor genesis. As a result, the main remedial target for PCa is reducing the levels of androgens (Zhou et al., 2015), which is accomplished through ADT. Despite this, severe complications are the resurrection of androgens and elevation of PSA. This condition is called CRPC and is characterized by a loss in the ability to respond to ADT, leading to reappearance of PCa and metastasis (Perlmutter and Lepor, 2007; Hotte and Saad, 2010). The translation of hormone-dependent PCa cells to CRPC is chiefly driven by the upregulation of AR activity. Urbanucci et al. have reported the overexpression of AR receptors in 22–30% of patients with CRPC (Urbanucci et al., 2011; Petrylak, 2013; Coutinho et al., 2016). In another study, Hay et al. have declared that the AR gene mutation was found in 22–30% of CRPC cases (Lobaccaro et al., 2012; Eisermann et al., 2013). Sharifi has shown the metabolic transformation of DHT (dihydrotestosterone). The USFDA has approved docetaxel for the management of CRPC. Similarly, cabazitaxel, another taxol derivative, has been tested in phase III clinical trial against CRPC. Numerous research groups have reported differentially expressed genes and phytochemicals established as inhibitors of identified targets for CRPC (Sharifi, 2013).

Rotimi et al. have conducted differential gene expression analysis of phytoconstituents target for CRPC. Plant-based phytomolecules were subjected to virtual screening toward GUCY1A2 variants. The results have revealed that SYT4, GUCY1A2, and GRIN3A were the most pharmacologically significant genes implicated in the pathogenesis of CRPC in the xenograft model (Rotimi et al., 2019). The docking scores of (8′S)-neochrome and (8′R)-neochrome were −152.102 and −160.75, respectively, when targeting G723S and Q217H. In another report, Cai et al. have observed an elevated expression of the α-subunit of soluble guanylyl cyclases (α-sGC) in hormone-refractory PCa at both the mRNA and protein levels (Krumenacker et al., 2004; Cai et al., 2006). It has been found that α-sGC leads to suppression of apoptosis via accumulation of p53 in the cytoplasm; hence, it is proposed as a target for CRPC. In another approach, Liao et al. have reported the screening of natural products, including terpenes, alkaloids, flavonoids, and polyphenols. Among them, rutaecarpine, obtained from *Tetradium ruticarpum* (A.Juss.) T.G.Hartley, has been found to exhibit potential effects against CRPC using *in vitro* and *in vivo* studies in LNCap and 22 RV1 cell lines and in xenograft models, respectively. The outcomes of western blotting analysis have revealed that AR-V7 and AR-FL were expressed in LNCaP and 22Rv1. Moreover, rutaecarpine has been found to exhibit a dose-dependent downregulated AR-V7 protein expression (Liao et al., 2020). Mendiratta et al. have established the genomic stratagem to manage CRPC using a transcription-based androgen receptor activity signature toward LNCaP cell lines (Mendiratta et al., 2009). AR signature has been employed to determine whether AR activity varies with hormone therapy; progression and oncogenic pathway assays were used to recognize biologic pathways associated with AR activity. The probability of AR activity was 0.13, 0.11, 0.96, and 087 against PC-3, DU-145, LNCaP, and 22Rv PCa cell lines, respectively (Amler et al., 2000; Febbo et al., 2005; Hieronymus et al., 2006). Moreover, these genomic targets served as diagnostic and prognostic biomarkers, and their discovery has been expedited multifold due to NGS technologies (Chen et al., 2013a; Chen et al., 2013b; Shen et al., 2021). Thus, discovering these biomarkers is fundamental for developing diagnostic biosensors (Jiang et al., 2014).

## Natural Products for the Treatment Management of Castration-Resistant Prostate Cancer

### Cheminformatics and Bioinformatics-Based Studies for Anti-Castration-Resistant Prostate Cancer Natural Products

#### Molecular Docking-Based Studies

Molecular modeling analyses are effective tools for studying structure–activity relationships (SARs). Several plant-based molecules have been screened to detect and identify various biological activities (Willett, 1994; Kumar and Jain, 2016; Sharma et al., 2016; Kumar et al., 2017; Kaur et al., 2019; Kumar et al., 2021). In the literature, numerous phytomolecules have been obtained from plants, such as alkaloids, tannins, glycosides, coumarins, flavonoids, and polyphenolic constituents, which have been evaluated against various cancer cell lines (Kumar D. et al., 2015; Kumar et al., 2016a; Kumar et al., 2016b; Kumar et al., 2018a; Kumar et al., 2018b; Kaur et al., 2020; Sharma et al., 2021). A wide range of natural products with high biocompatibility, low toxicity, good sustainability, and a good safety profile is extensively used to manage CRPC.

#### Structure–Activity Relationship and Mechanistic Insights

**FIGURE 3:**
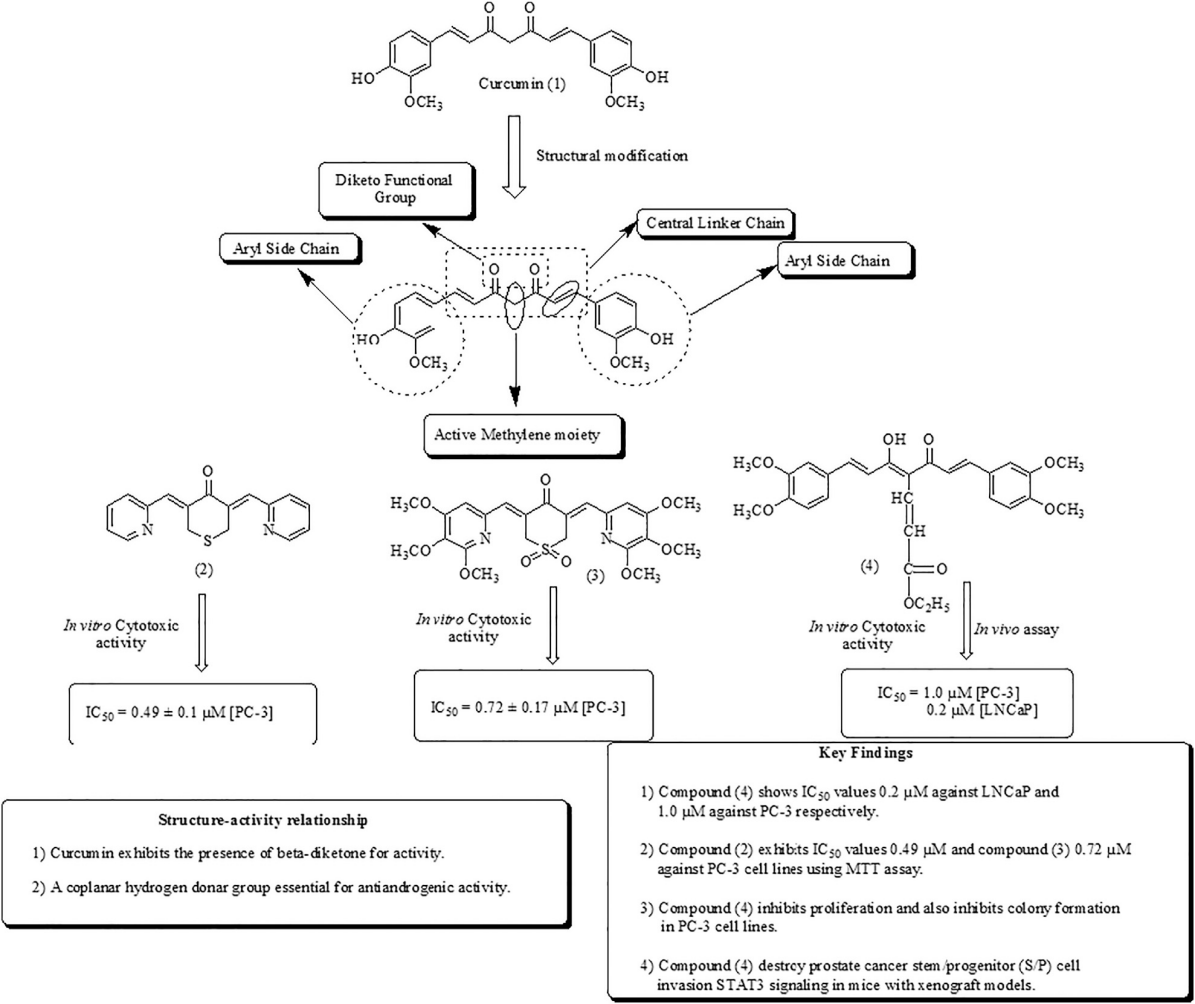
Structure of curcumin along with mechanistic insights.

Several phytoconstituents have been isolated from various plants and screened for *in vitro, in vivo, in silico,* and mechanistic insights into mitigating CRPC. Mbese et al. have reported the therapeutic role of curcumin and its derivatives in treating PCa (Mbese et al., 2019). Curcumin is one of the main constituents of *Curcuma longa* L. (Zingiberaceae). It is a polyphenolic compound widely used as a pigment and spice, available in the market as turmeric and commonly known as Haldi. Curcumin structure and its most potent analogs (1–4) are shown in Figure 3 (Mukhopadhyay et al., 2001; Yang et al., 2006; Choi et al., 2010; Yallapu et al., 2014; Schmidt and Figg, 2016).

**FIGURE 4:**
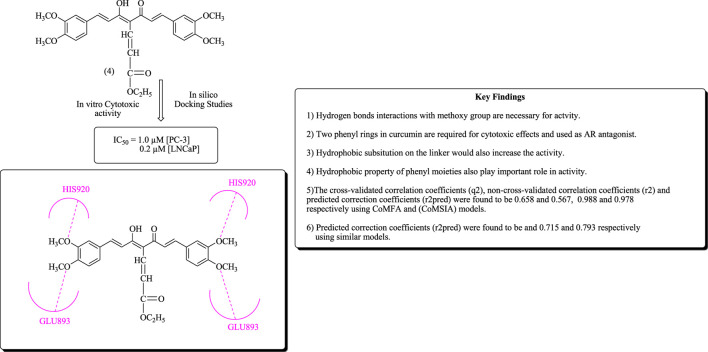
Binding interactions of compound (4) with amino acid residues along with important key findings.

Xu et al. have reported the docking studies and three-dimensional quantitative SARs of curcumin analogs as androgen receptor antagonists. The bioactive conformation was explored using molecular docking in SYBYL with binding interaction of AR. The oxygen atom of the methoxy group forms binding interactions as a hydrogen bond acceptor by creating hydrogen bonds with HIS920 and GLU893, respectively, as depicted in Figure 4 (Cramer et al., 1988; Lill, 2007; Xu et al., 2012).

**FIGURE 5:**
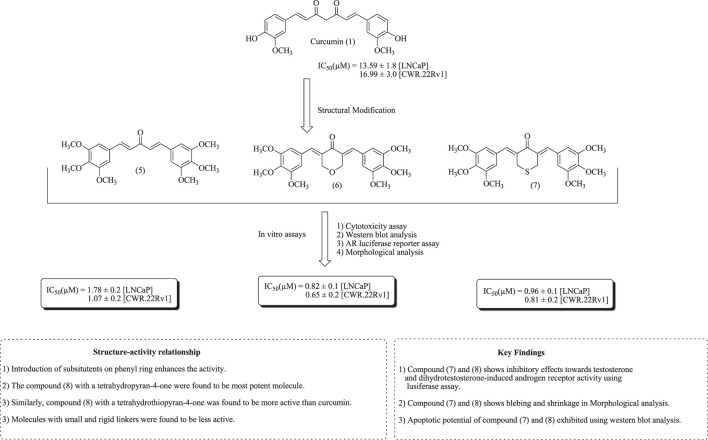
Structure of curcumin and its analogs (5–7) along with structure–activity relationships and mechanistic insights.

Zhou et al. have established the role of curcumin and its analogs in androgen receptor activation and inhibiting the growth of human PCa, such as LNCaP and CWR-22Rv1 cell lines. SAR and the apoptotic effect of curcumin and its analogs (5–7) are presented in Figure 5 (Zhou et al., 2014).

**FIGURE 6:**
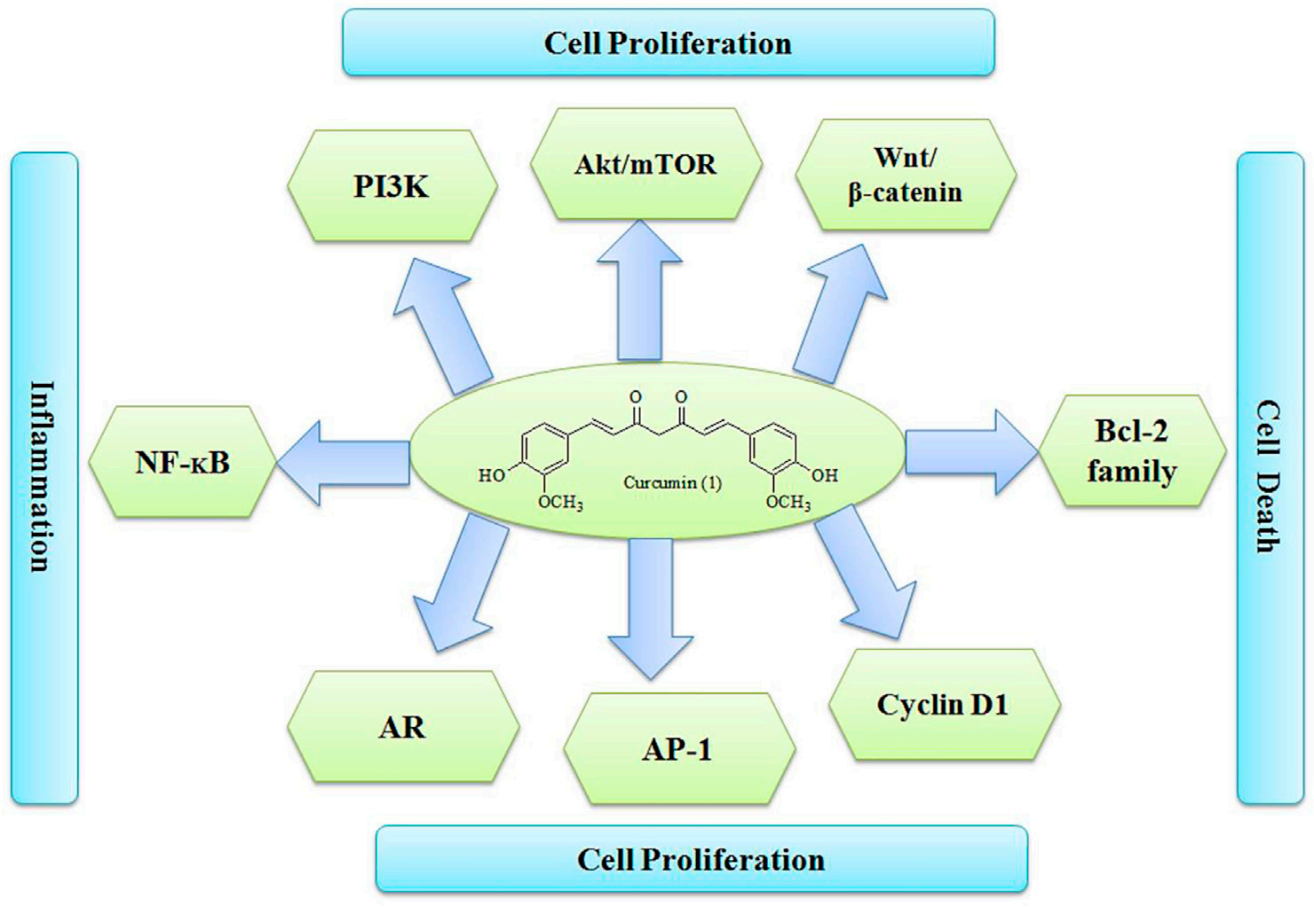
Molecular targets of curcumin.

Numerous research groups have established molecular targets of curcumin (1) associated with cell proliferation, cell death, and inflammation. Androgen receptor signaling, activation of protein-1, PI3K/Akt/mTOR, Bcl-2 family, NF-_K_B, and wingless β-catenin signaling are shown in Figure 6, along with important mechanistic insights (Abd. Wahab et al., 2020). Curcumin can also be recommended along with prednisone and docetaxel in individuals with CRPC. It can also be given in combination with isoflavones in patients who had prostate biopsy due to elevated PSA levels (Ide et al., 2010; Mahammedi et al., 2016).

**FIGURE 7:**
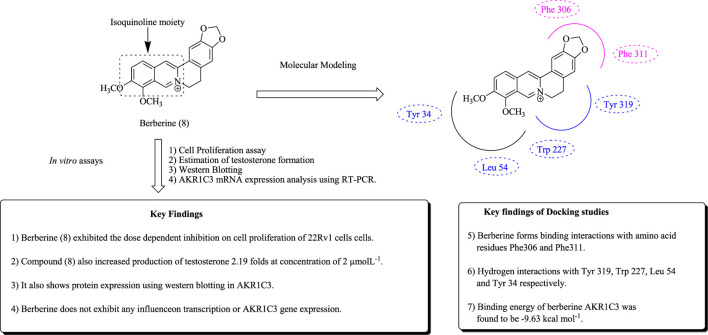
Structure of berberine along with important key findings.

Berberine (8) is an isoquinoline derivative and has been evaluated for anticancer activities against PCa (Li et al., 2011; Wang et al., 2012). Tian et al. have reported the effect of berberine in inhibiting synthesis, which employs the interaction with aldo-keto reductase 1C3 as a potential target against 22Rv1 prostate cancerous cell lines. Berberine delayed the progression of CRPC by reducing androgen synthesis. Moreover, protein levels were determined using western blotting and RT-PCR, respectively (Li et al., 2016). Molecular docking studies of berberine are shown in Figure 7. In another study, Mantena et al. have reported the apoptotic potential of berberine; that is, berberine induces cell cycle arrest in the G1 phase and caspase-3 inhibition in human PCa cell lines (Mantena et al., 2006).

**FIGURE 8:**
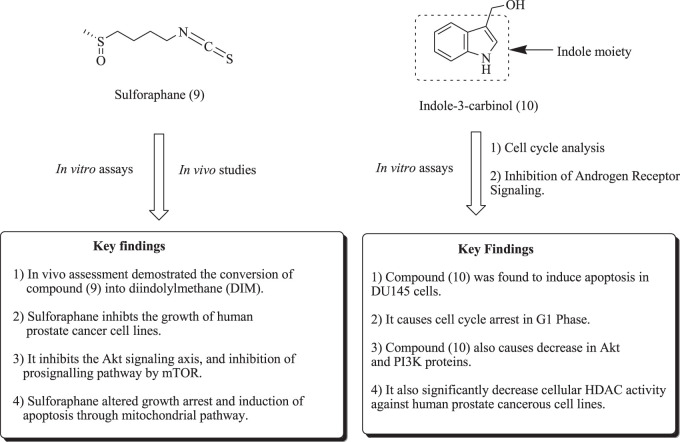
Structures of sulforaphane and indole-3-carbinol along with mechanistic insights.

In another report, Watson et al. have identified the secondary metabolites produced from cruciferous vegetables, such as broccoli, cauliflower, and brussels sprouts. These metabolic products, such as sulforaphane (9) and indole-3-carbinol (10), were potential candidates for inhibiting PCa as an epigenetic modulator. Structures of sulforaphane and indole-3-carbinol are depicted in Figure 8 along with mechanistic insights (Chinni et al., 2001; Li, 2005; Garikapaty et al., 2006; Beaver et al., 2012; W.; Watson et al., 2013).

**FIGURE 9:**
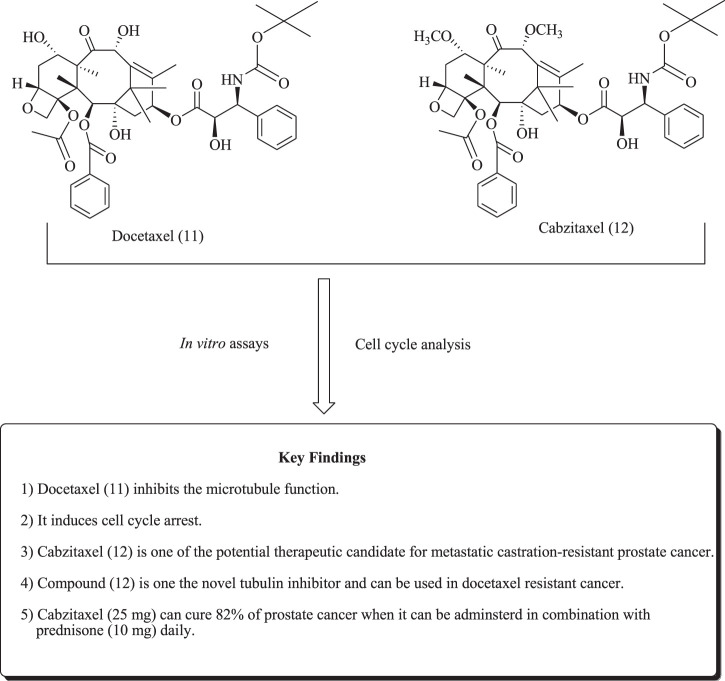
Structure of docetaxel and cabazitaxel.

Docetaxel (11) is a semisynthetic taxane derivative and is widely used to mitigate numerous cancers, such as prostate, breast, ovarian, lung, and pancreatic cancers (Oudard et al., 2017). Similarly, cabazitaxel (12) is a second-generation docetaxel derivative with activity against docetaxel-resistant tumors. Compound (12) exerts its action by inhibiting microtubule functions in PCa cell lines (Kotsakis et al., 2016). Bono et al. have reported the use of cabazitaxel in combination with prednisone in metastatic CRPC (de Bono et al., 2010). The structure of taxanes derivatives is depicted in Figure 9.

**FIGURE 10:**
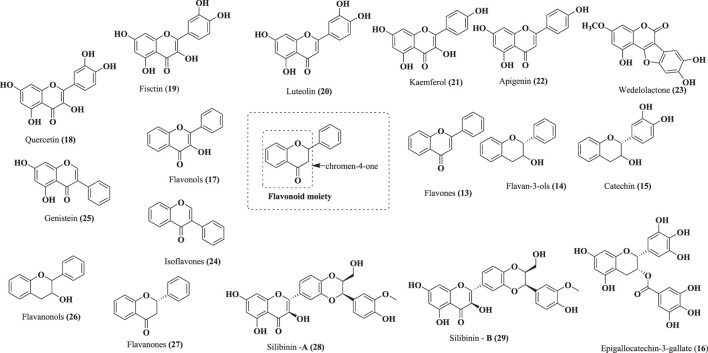
Structures of flavonoid-based phytochemicals (13–29).

Flavonoid-based phytochemicals are characterized by a 15-carbon frame as a common phenyl benzopyrone association (C_6_–C_3_–C_6_) in their scaffolds (Sharma et al., 2021). Flavonoids are a potential bioactive class of natural products and are subdivided into flavones, flavanones, flavan-3-ols, flavonols, flavanones, and isoflavones. Among these, phytomolecules, quercetin, kaempferol, luteolin, apigenin, genistein, fisetin, epigallocatechin-3-gallate, and a mixture of flavo-lignans, such as silibinin-A and silibinin-B, have been screened against CRPC using *in vitro*, *in vivo,* and preclinical studies (Kallifatidis et al., 2016; Taylor and Jabbarzadeh, 2017; Salehi et al., 2019; Fontana et al., 2020). Lin et al. have isolated wedelolactone from *Sphagneticola calendulacea* (L.) Pruski and evaluated its effect on the growth of PCa cell lines, such as 22Rv1 and LNCaP, with IC_50_ values of 0.4 µg/ml and 0.8 µg/ml, respectively (Lin et al., 2007). Structures of flavonoid-based phytochemicals (13–29) are shown in Figure 10.

**FIGURE 11:**
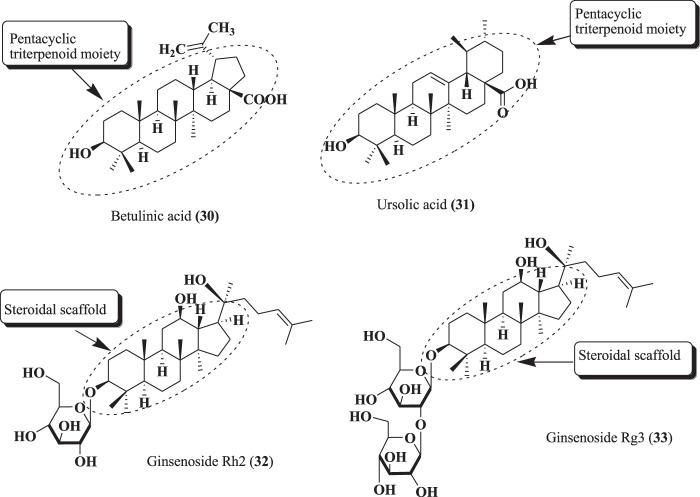
Structure of compounds (30–33).

Various pentacyclic triterpenoids and steroid-based phytochemicals were found to be potential candidates for the treatment of CRPC (Fulda, 2008; Reiner et al., 2013). Among these, betulinic acid (30), ursolic acid (31), ginsenoside Rh2 (32), and ginsenoside Rh3 (33), as depicted in Figure 11, exhibited remarkable effects against PCa (Kallifatidis et al., 2016).

**FIGURE 12:**
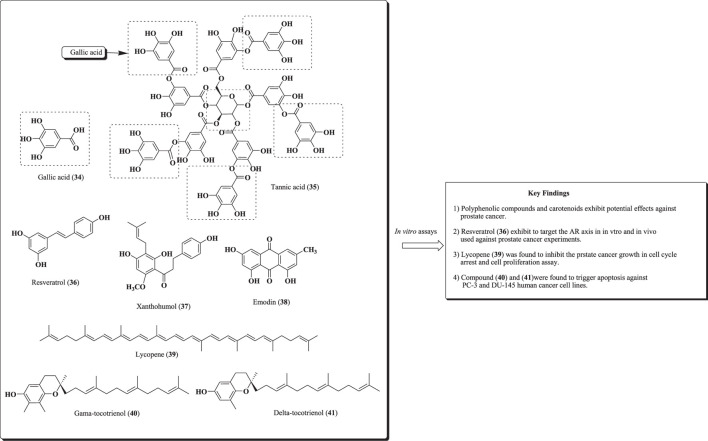
Structure of compounds (34–41) along with important key findings.

Mokbel et al. have reported the phytomolecules obtained from dietary components, which exhibited remarkable effects against PCa. The effects of these compounds have been revealed using *in vitro* assays, cell proliferation assay, and cell cycle arrest studies in PCa cell lines (Fontana et al., 2019a; Fontana et al., 2019b; Luo et al., 2019; Mokbel et al., 2019; Montagnani Marelli et al., 2019). Structures of the compounds (34–41) are shown in Figure 12.

#### Machine Learning-Based Studies

Deep learning and machine learning have been implemented in various drug discovery processes such as physiochemical activity, pharmacophore modeling, QSAR, toxicity prediction, and structure based-virtual screening (Mishra et al., 2017; Joon et al., 2021). With the recent advancement in modern technologies, integrated artificial intelligence algorithms and computer-aided drug design can help overcome the hurdles and challenges of conventional drug design (Duch et al., 2007; Lipinski et al., 2019). Badillo et al. have reported the advancements in predicting biomarkers using machine learning to provide physicians with new insights into diagnosis. Using deep machine learning, the application of biomarkers such as prostate-specific antigen (PSA) and its clinical relevance in the prediction of metastasis of PCa are examined. One of the primary challenges in PCa management is deciding which patients have clinically significant tumors. This concern includes not only new patients but also relapsed patients after primary treatment (Majumdar, 1985; Zhang et al., 2017; Badillo et al., 2020; Gupta et al., 2021).

In another study*,* Pantuck et al. have developed CURATE.AI to determine adequate drug doses. In this study, a combination of enzalutamide and experimental drug ZEN-3694 was administered to a patient with metastatic CRPC. Using CURATE.AI, it was found that a dose 50% lower than the starting dose of ZEN-3694 can achieve the desired results and arrest PCa growth (Pantuck et al., 2018; Su et al., 2019). Similarly, Kaiwen Deng et al. have reported treating patients with mCRPC by machine learning using a computational model. Through this model, patients were accurately allocated to docetaxel-intolerable and docetaxel-tolerable groups. This algorithm predicts the adverse effects of docetaxel treatment in patients. In this experiment, the data were collected from 1600 patients in phase III clinical trials for PCa treatment. These data generated the gold standards framework, including treatment status, discontinuation, and the number of deaths. The discontinuation status can be envisaged using models with clinical parameters. Moreover, death and treatment status were associated with discontinuation (Deng et al., 2020).

Lee et al. have developed the novel machine learning model for investigating non-mCRPC. In this model (Survival Quilts), an algorithm automatically tunes and selects ensembles of survival models based on clinical-pathological parameters using the Surveillance, Epidemiology, and End Results (SEER) datasheet. Data have been collected from (approximately) 30% of the US population, especially from men aged 35–95 years (Adamo et al., 2016; Lee et al., 2021). Survival Quilts is open-source software designed to mechanize the operation of machine learning in estimating survival rates. Demographic characteristics of patients were age, T-stage, PSA, primary and secondary Gleason grades score or grade groups, PCa-specific mortality, and all-cause mortalities. This machine learning model is competent in predicting ten-year PCa-specific mortality (Lee C. et al., 2019).

**FIGURE 13:**
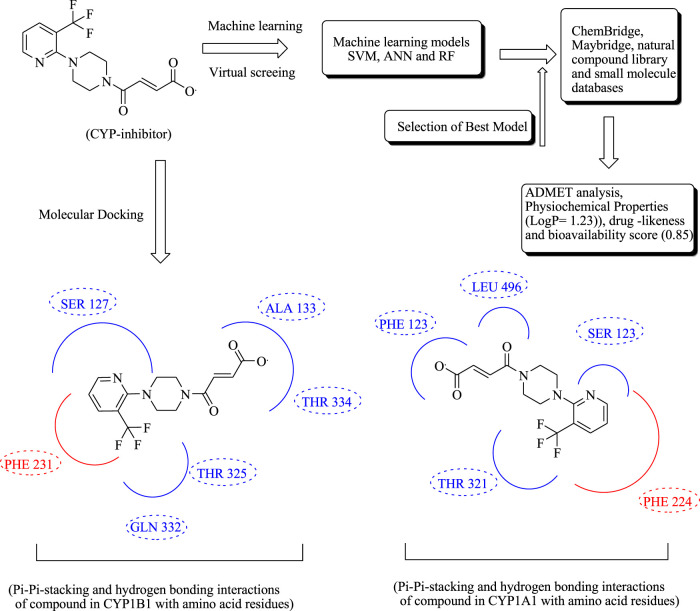
Schematic representation of machine learning and molecular docking of CYP inhibitors.

In another study, Raju et al. have conducted multiple machine learning, ADMET screening, and molecular docking studies to identify selective inhibitors of CYP1B1. Different machine learning models have been developed along with molecular databases, including Maybridge, ChemBridge, and a natural compound library, from which the selected models of CYP1B1 and CYP1A1 were evaluated. These inhibitors were highly expressed in wide varieties of cancer such as prostate, colon, and breast. The most widely used anticancer compounds, including paclitaxel, tamoxifen, docetaxel, and imatinib, are rapidly inactivated by CYP1B1, eventually leading to drug resistance (Androutsopoulos et al., 2009; Afarinkia et al., 2013; Raju et al., 2021). These inhibitors were subjected to molecular docking and pharmacokinetic analysis, as shown in Figure 13.

#### Role of Network Pharmacology Approach in Castration-Resistant Prostate Cancer

**FIGURE 14:**
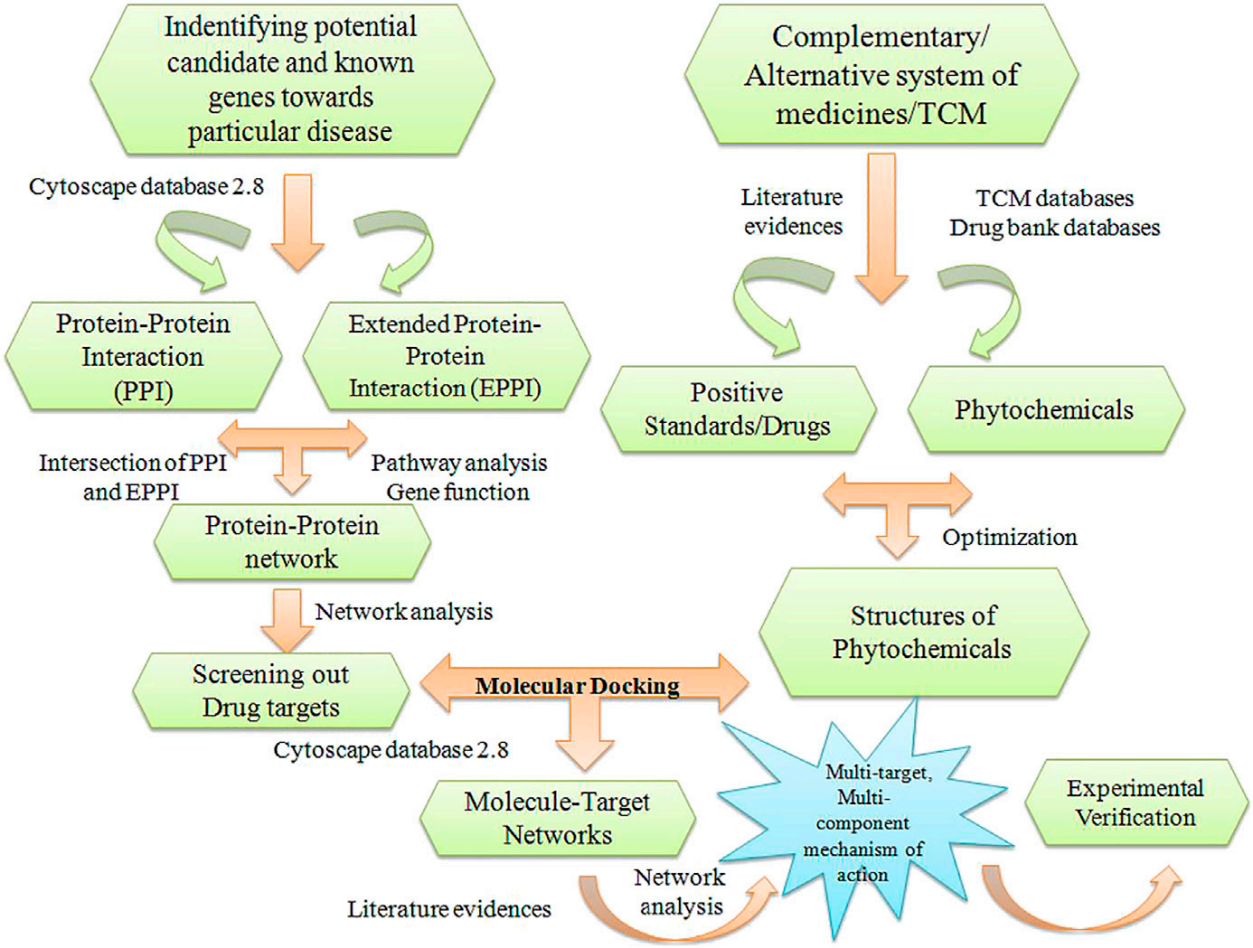
Schematic representation of network pharmacology framework.

Traditional Chinese systems of medicine and complementary and alternative systems of medicine are the three main systems used to manage many types of cancer such as colon, gastric, breast, ovarian, and prostate cancer. The mechanisms of phytomolecules against CRPC have not been fully explored in the literature (Chen et al., 1996; Liu et al., 2018). Various research groups have reported the network pharmacology-based approach to explore natural products such as curcumin, quercetin, and ursolic acid as potential candidates for the treatment of CRPC (Barton et al., 2008; Hopkins, 2008; Li et al., 2014; Ru et al., 2014; Lee D. et al., 2019). Li et al. have demonstrated the network pharmacology of TCM based on the binding interactions of natural herbs, isolated phytochemicals, targets, genes, and diseases (Li et al., 2010; Li and Zhang, 2013). The main goal of network pharmacology is to investigate a potential candidate against disease with high efficiency, minimal side effects, and less toxicity. Song et al. have developed a network pharmacology-based technique to explore the mechanism of *Scleromitrion diffusum* (Willd.) R.J.Wang as a therapeutic candidate against CRPC. Prospective target genes of PCa were screened using databases such as OMIM, DisGeNET, and GeneCards. A network was constructed by evaluating the possible interactions among diverse target nodes. Protein–protein interaction, Kyoto Encyclopedia of Genes and Genomes (KEGG), and Gene Oncology enrichment analyses have been performed to explore and discover the mechanistic insights and pathways for therapy against PCa (Zhu et al., 2014; Yu et al., 2020). The PPI network revealed the multiple imperative targets and PCa-related targets, such as PI3K, AkT1, mTOR, BCL2, Cyclin D1, PARP1, MAPK1, MMP3, MMP9, caspase-3, caspase-9, STAT-3, and RAF-1 (Song et al., 2019). A general schematic representation of the network pharmacology framework is depicted in Figure 14 (de Carvalho et al., 2014).

Bi et al. have established the mechanism of curcumin against tumors based on pharmacology networking using DAVID 6.8 and GeneMANIA server database for analysis. This study identified multiple drug targets, target pathways, and signaling pathways (Bi et al., 2018). Thus, phytomolecules can also be used in the future to manage other diseases, as stated in complementary and traditional systems of medicine (Choi et al., 2010).

### Translational Studies of Phytomolecules for Castration-Resistant Prostate Cancer: Assessment of Translation Potential for Bench to Bedside

Over the last 40 years, most chemotherapy drugs used to treat cancer originated from natural products. Moreover, phytomolecules may provide various lead structures used as templates to synthesize new, pharmacologically more effective agents (Mann, 2002). Since concomitant side effects, if any, are reported to be mild, plants and their metabolites are considered to be potentially acceptable choices for chemoprevention. It has been shown that several phytocompounds, such as genistein, green tea polyphenols, curcumin, lycopene, and vitamin D, prevent or postpone the onset of PCa or its development to CRPC (Kallifatidis et al., 2016). Basic and applied research are two types of study that are usually separated. Basic research is important for better understanding normal and pathological states, but it does not directly translate this information into therapeutically relevant applications. Based on our understanding of illness formation and progression, applied research aids in the development of novel diagnostic tools or treatments for patients. The major objective of “translational” research is to combine molecular biology advances with clinical trials, bringing research from the bench to the patients’ bedside (Gibbs, 2000; Saijo et al., 2003; Goldblatt and Lee, 2010). Most cancers likely develop due to multiple genetic abnormalities, implying the need for a cocktail of agents against multiple targets in cancer cells or the use of agents with a broad range of targets, which is one of the most critical factors limiting the effectiveness of targeted therapy. The treatment of CRPC patients is still a serious clinical issue. Researchers now have a better knowledge of the mechanisms of CRPC owing to molecular, basic, and translational studies (Amaral et al., 2012). Many clinical trials to assess the efficacy of phytochemicals in human subjects have been undertaken, and they have partially corroborated the encouraging results found *in vitro* and preclinical models. We will only discuss those clinical studies where the pure phytocompounds or the characterized phytofractions were considered. We have excluded studies on extracts and juices, as they would have deviated content from the theme. Plant-derived constituents showing remarkable anticancer effects against CRPC in clinical trials are discussed as follows.

*Resveratrol*. Resveratrol is a polyphenol found in nature that has been shown to inhibit PCa growth and development. In several preclinical investigations, resveratrol has been shown to decrease prostate cancer development *in vitro* and in animal models. Moreover, resveratrol has been found to reduce androgen receptor expression, decrease proliferation, induce apoptosis in PCa cell lines, and improve their ionizing radiation sensitivity (Jasinski et al., 2013). Dietary resveratrol suppresses β-catenin-mediated AR signaling and represses nuclear localization of β-catenin by reducing HIF-1 production, perhaps in a proteasome-independent way, contributing to the reduction of CRPC tumor progression (Mitani et al., 2014). Kjaer et al. have conducted a randomized controlled trial (RCT) using two doses of resveratrol (150 mg or 1,000 mg resveratrol daily) for 4 months in 66 middle-aged men suffering from metabolic syndrome. High-dose resveratrol (1,000 mg daily) treatment for 4 months dramatically reduced blood levels of the androgen precursors androstenedione, dehydroepiandrosterone, and dehydroepiandrosterone sulfate. However, the prostate size and circulating levels of PSA, testosterone, free testosterone, and dihydrotestosterone were unchanged (Kjaer et al., 2015). Patients with nonmetastatic biochemically recurrent PCa were allocated to escalating doses of MPX (pulverized muscadine grape skin rich in ellagic acid, quercetin, and resveratrol) in cohorts of two patients, with six patients at the maximum dose in the phase I section of this phase I/II trial, which used a modified continuous reassessment technique. The phase I section revealed that the largest dose tested, 4,000 mg/d, was safe, with only grade 1 adverse event being recorded. Even though the phase I population was small and there was no sustained decrease in serum PSA from baseline, the findings show that 4,000 mg/d of muscadine grape skin extract is safe (Paller et al., 2015).

Furthermore, Paller et al. have conducted a randomized, multicenter, placebo-controlled, dose-evaluating phase II trial. The results on 112 biochemically recurrent (BCR) patients were evaluated, revealing that MPX did not significantly improve the prostate-specific antigen doubling time (PSADT) compared with the placebo. Nevertheless, other benefits have been observed in the exploratory analysis (Paller et al., 2018). Further, testing resveratrol’s chemopreventive effects in conjunction with other antioxidants occurring naturally together, such as in grapes, might be beneficial (Singh et al., 2016).

*Soy Isoflavone*. Incidence rates of PCa are lowest in Asian nations, where soy foods are frequently part of a normal diet (Jian, 2009). In some animal models, physiologically active isoflavones found in soy products, such as genistein, daidzein, equol, and glycitin, prevented PCa (Mahmoud et al., 2014). Antioxidant defense, DNA repair, suppression of angiogenesis and metastasis, potentiation of radio- and chemotherapeutic drugs, and antagonism of estrogen- and androgen-mediated signaling pathways are all involved in soy isoflavone-induced growth arrest and death of PCa cells (Mahmoud et al., 2014). In numerous clinical trials (Table 1), soy isoflavones have been found to reduce PSA levels.

**TABLE 1:** Clinical studies evaluating the potential of soy isoflavones in reducing PSA levels.

Clinical study protocol	Outcome	PMIDs	References
Healthy men (*N* = 112 aged 50–80 years) were randomly allocated to groups drinking either a soy protein drink with 83 mg of isoflavones (+ISO) or a comparable drink with isoflavones removed in a double-blind, parallel-arm, randomized experiment for 12 months	In the isoflavone therapy group, there was no significant change in blood PSA level, velocity, or PCa incidence	15066931	Adams et al. (2004)
For 3 months, 24 males were put on a high or low soy diet in a randomized, double-blind, crossover clinical trial	14% decrease in circulating blood PSA levels was observed but with no change in testosterone levels	16775579	Maskarinec et al. (2006)
In a randomized study, 58 men at high risk of PCa were allocated to groups taking one of three protein isolates containing 40 g/d protein at random (107 mg/d isoflavones, <6 mg/d isoflavones, or 0 mg/d isoflavones) for 6 months	Soy protein isolate intake reduces AR expression in the prostate but did not affect ER β expression	17585029	Hamilton-Reeves et al. (2007)
Twenty patients with increasing PSA following previous local treatment were treated with soy milk with 47 mg of isoflavonoid per 8 oz serving three times per day for 12 months in an open-labeled phase II study	In six patients, the slope of PSA after study enrollment was substantially lower than that before entering the study, while in two individuals, the slope of PSA after study admission was significantly greater	18471323	Pendleton et al. (2008)
In a randomized, double-blind experiment, 25 PCa patients were given placebo or soy isoflavone supplements for 2 weeks before prostatectomy	In PCa patients, soy isoflavones decreased prostate COX-2 mRNA while increasing p21 mRNA	19127598	Swami et al. (2009)
In the phase II trial, 29 patients with increasing PSA levels following intense radiotherapy for prostate cancer were told to drink 500 ml of soy beverage every day for 6 months	In 41% of PCa patients, soy caused a substantial delay in PSA doubling time	20099194	Kwan et al. (2010)
53 men with PCa took a daily supplement comprising 450 mg genistein, 300 mg daidzein, and other isoflavones for 6 months in a double-blind, placebo-controlled, randomized study	In men with low-volume PCa, there was no significant reduction in PSA levels	21058191	deVere White et al. (2010)
33 males undergoing androgen deprivation therapy for PC were given either 20 g of soy protein with 160 mg of total isoflavones or a taste-matched placebo (20 g whole milk protein) for a 12-weeks	In androgen-deficient males, high-dose isoflavones do not enhance metabolic or inflammatory markers	20798386	Napora et al. (2011)
Phase II, randomized, double-blind, placebo-controlled trial, or oral isoflavone (60 mg/day) for 12 months, *N* = 158	PSA levels did not significantly change, following treatment with isoflavones. The isoflavone group had a substantially reduced PCa incidence in 53 individuals aged 65 years	21988617	Miyanaga et al. (2012)
47 Norwegian patients were given 30 mg genistein or placebo capsules daily for 3–6 weeks before prostatectomy in a phase 2 placebo-controlled, randomized, double-blind clinical study	mRNA level of KLK4 in tumor cells was considerably decreased, while androgen and cell cycle-related biomarkers were not significantly lowered	22397815	Lazarevic et al. (2012)
A double-blinded, randomized, placebo-controlled trial included 86 men given soy isoflavone capsules (80 mg/d of total isoflavones and 51 mg/d aglucon units) for 6 weeks	After consuming soy isoflavones for a short time, no significant changes in blood hormone levels, total cholesterol, or PSA were observed	`23874588	Hamilton-Reeves et al. (2013)
In 177 men at high risk of recurrence following radical prostatectomy for PCa, a randomized, double-blind trial comparing daily use of beverage powder containing 20 g of protein in the form of either soy protein isolate (*n* = 87) or as placebo calcium caseinate (*n* = 90). Within 4 months of surgery, supplementation was started and was followed up for 2 years	Following radical prostatectomy, daily intake of a beverage powder supplement containing soy protein isolate for 2 years did not prevent biochemical recurrence of PCa in men at high risk of PSA failure	23839751	Bosland et al. (2013)

*Lycopene*. Lycopene, a natural pigment found mostly in the ripe tomato fruits, is gaining popularity in preventing and treating heart disease and cancer (Heber and Lu, 2002). Lycopene’s anticancer actions on PCa cells are mediated via decreasing cell proliferation, inducing apoptosis, stopping the cell cycle, and lowering DNA damage in various investigations (Ivanov et al., 2007; Holzapfel et al., 2013). *In vitro* studies have shown that a normal physiological concentration of lycopene in culture conditions inhibits the growth of PCa cell lines, which are either androgen-dependent or androgen-independent (Kim et al., 2002; Mirahmadi et al., 2020). A human intervention study has demonstrated that consumption of carotenoid-containing plant products significantly decreased oxidative base damage (Pool-Zobel et al., 1997). In phase II randomized clinical trial, 26 men with newly diagnosed, clinically localized PCa randomly allocated to groups taking 15 mg of lycopene twice daily (*n* = 15) or no supplementation (*n* = 11) for 3 weeks before radical prostatectomy exhibited reduced PSA, connexin 43, and insulin-like growth factor-1 levels (Kucuk et al., 2001). In an unblinded randomized phase I clinical study, 32 patients with localized prostate adenocarcinoma consumed tomato sauce-based pasta dishes for 3 weeks (30 mg lycopene/day), which resulted in higher lycopene levels in prostate tissues and serum, reduction in PSA serum levels, and DNA damage in both prostate and leukocyte cells in prostatectomy (Bowen et al., 2002). In a prospective trial, 20 metastatic HRPC patients were given 10 mg lycopene daily for 3 months and in almost all men, disease progression inversely changed to a lower grade. Moreover, PSA levels were reduced and ECOG function and bone pain improved (Ansari and Gupta, 2004). In phase II clinical trial, 10 mg lycopene per day for 1 year resulted in a reduced PSA velocity in 40 individuals (Barber et al., 2006). Bunker et al. have conducted a randomized, unblinded phase I clinical trial in 81 high-risk Afro-Caribbean patients with neoplasia and found that 30 mg/day of lycopene for 4 months lowers PSA serum concentrations (Bunker et al., 2007). In a phase II research, 46 androgen-independent PC patients were given lycopene (15 mg twice daily), which resulted in a 50% drop in PSA levels; however, the trial showed no clinical benefit for the advanced stage of cancer (Jatoi et al., 2007). In another trial, 32 patients with high-grade prostatic intraepithelial neoplasia were given a lycopene-enriched diet (20–25 mg/day) for 6 months; however, no meaningful effect was observed in terms of lycopene’s effects on cancer development or PSA levels in these individuals (Mariani et al., 2014). In phase II research, Zhuang et al. have studied the clinical efficacy and safety profile of docetaxel with lycopene in CRPC patients and revealed that the combination of docetaxel and lycopene resulted in enhanced PSA response rate and tolerability (Zhuang et al., 2021).

*Quercetin*. Quercetin, a flavonoid found in fruits and vegetables, has been shown to have anti-inflammatory, antioxidant, and cancer-fighting properties. The activity of promoters of two major genes implicated in PCa pathogenesis, i.e., AR and PSA, is inhibited by quercetin (Ghafouri-Fard et al., 2021). By suppressing the main survival protein Akt, quercetin has also been shown to promote the apoptosis of PCa dose-dependently (Ghafouri-Fard et al., 2021). On PC-3 cells (model cells of CRPC), quercetin and paclitaxel coadministration significantly reduced cell proliferation, increased apoptosis, triggered cell cycle arrest at the G2/M stage, activated endoplasmic reticulum stress, and enhanced reactive oxygen species production (Zhang X. et al., 2020). Henning et al. have conducted a prospective randomized, parallel design, placebo-controlled trial in which 31 men with PCa were given either 1 g of green tea extract containing 830 mg of green tea polyphenols with 800 mg of quercetin or placebo for 4 weeks before prostatectomy (Henning et al., 2020). Following the coadministration of green tea extract and quercetin, they have found a significant rise in quercetin concentrations in plasma, urine, and prostate tissue. Furthermore, this regimen decreased epicatechin gallate levels in the urine. In prostate tissue or RBCs, no significant change in the concentration of green tea polyphenols or methylation activity across the groups was observed and there was no evidence of liver injury (Henning et al., 2020).

*Gossypol*. (-)-Gossypol, a polyphenolic chemical found in cottonseed, improves radiation therapy response and shrinks human PCa tumors (Xu et al., 2005). (-)-Gossypol induced apoptosis in DU-145 cells by downregulating Bcl-2 and Bcl-xL and increasing Bax at the mRNA and protein levels. It also enhances PARP cleavage and activates caspase-3, -8, and -9 (Huang et al., 2006). In PCa cells and prostate tumor-initiating cells, gossypol activates p53 and induces apoptosis (Volate et al., 2010). AT-101 (R-(-)-gossypol acetic acid; Ascenta Therapeutics, Inc.), a derivative of gossypol, exhibited anticancer activity in various tumor models. Liu et al. have conducted a phase I/II study of AT-101 in 23 patients with CRPC and investigated that AT-101 was well tolerated when given at a dose of 20 mg/day for 28 days (Liu et al., 2009).

*(-)-Epigallocatechin-3-gallate (EGCG)*. (-)-Epigallocatechin-3-gallate (EGCG), biologically active catechin of green tea, has been evaluated for its chemopreventive activity against CRPC using *in vitro* and *in vivo* animal studies (Ju et al., 2005; Thangapazham et al., 2007; Rahmani et al., 2015). Bettuzzi et al. have explored cancer-preventive effects of green tea catechins (GTCs) in volunteers with high-grade prostatic intraepithelial neoplasia (HGPIN). The volunteers were given 200 mg of GTC three times daily for a total of 600 mg/d and followed up after 1 year. Only one instance of PCa in the treatment group (incidence 3%) and nine cases of PCa (incidence 30%) in the placebo group were observed (Bettuzzi et al., 2006). A decrease in PSA levels, although not significant, and a significant decrease in International Prostate Symptom Score were observed in the GTC group. Furthermore, during a 2-year follow-up, two of the nine placebo males and one of the 13 GTC patients were diagnosed with PCa, demonstrating an 80% reduction in PCa diagnosis in patients with HGPIN (Brausi et al., 2008). Polyphenon E (PolyE), a mix of GTCs, containing 400 mg of EGCG, was given daily for 1 year to men with HGPIN in a placebo-controlled, randomized clinical trial (Kumar NB. et al., 2015). A decrease in serum PSA levels and ASAP in the PolyE group was observed. In another study, males with PCa were given 1.3 g of PolyE containing 800 mg of EGCG daily (McLarty et al., 2009). A significant reduction in PSA, HGF, and VEGF serum levels was observed at the time of prostatectomy (after 3–6 weeks). Moreover, supplementation with PolyE containing 800 mg of EGCG for 3–6 weeks resulted in a beneficial but nonsignificant reduction in serum PSA in a similar clinical study (Nguyen et al., 2012). Contrary to the above observations, minimal antineoplastic action was identified after daily dosages of EGCG were given to 42 patients with androgen-independent PCa in phase II clinical study (Jatoi et al., 2003).

*Curcumin*. Curcumin, diferuloylmethane, inhibits PCa proliferation and metastatic development by downregulating androgen receptor and epidermal growth factor receptors and causing cell cycle arrest (Teiten et al., 2010). Twenty-six patients with advanced CRPC and elevated PSA were given docetaxel/prednisone in usual settings for six cycles, along with curcumin (6,000 mg per os each day; day −4 to day +2 of docetaxel) (Mahammedi et al., 2016). This study demonstrates that curcumin has a high response rate, good tolerability, and patient acceptability, justifying the need for a randomized trial. An RCT was conducted on 64 eligible patients with PCa to assess the beneficial role of nanocurcumin in preventing and/or mitigating radiation-induced proctitis in PCa patients undergoing RT (Saadipoor et al., 2019). 33 patients received nanocurcumin (120 mg/day) 3 days before and during the RT course. Radiation-induced proctitis occurred in 18/31 (58.1%) of placebo-treated patients compared to 15/33 (45.5%) of nanocurcumin-treated patients (Saadipoor et al., 2019). The role of anticancer effects of curcumin in patients with PCa that undergo intermittent androgen deprivation (IAD) treatment has been studied. A randomized, double-blind, placebo-controlled trial was performed on 97 patients with PCa (49 patients took oral curcumin (1440 mg/day) and 48 received placebo for 6 months) who received IAD treatment (Choi et al., 2019). The results have demonstrated that oral administration of curcumin for 6 months can lower PSA levels significantly in patients compared to those in the placebo group. Curcumin (total 3 g/day), a radiosensitizing and radioprotective agent, showed significant improvement of antioxidant status in patients with PCa who received radiotherapy (Hejazi et al., 2016). Another clinical study has been conducted to examine the effect of curcumin supplements and isoflavones on serum PSA levels (Ide et al., 2010). The isoflavones and curcumin were administered orally to patients who had prostate biopsy due to elevated PSA levels for 6 months, and a significant decrease of serum PSA level was observed. Two clinical studies on the effect of adjuvant use of curcumin after prostatectomy on improving recurrence-free survival in PCa patients (clinicaltrials.gov; NCT02064673) and reducing the risk of PCa progression in low-risk men (clinicaltrials.gov; NCT03769766) are undergoing.

## Conclusion and Future Perspectives

PCa is a leading cause of cancer-related morbidity and mortality. CRPC poses a pathophysiological and therapeutic challenge that imposes a significant burden on individuals and the healthcare system (Tang et al., 2013). A growing number of studies have focused on deciphering its mechanistic underpinnings and targeting the key elements of its pathogenesis. Therefore, it might be more effective to address several pathogenetic mechanisms contributing simultaneously to castration resistance and cancer progression. These mechanisms can also complement conventional and novel anticancer treatments. The main findings of our review can be summarized as follows:

• The tumor microenvironment, with its immune cells, cytokines, chemokines, androgen receptors and their molecular interactions, plays an important role in castration resistance. Simulating this environment can be a key to the success of cheminformatics modeling studies.
• Multiple signaling pathways and transcription factors are associated with CRPC. Natural compounds may be used to target their elements and disrupt them. However, this may be ground for potential side effects stemming from other body systems.
• Oncogenes and mutations (SYT4, GUCY1A2, GRIN3A, and BRAC) that are implicated in PCa resistance constitute therapeutic targets. Exploring the effect of natural products alongside the genetic traits of CRPC can lead to more precise and, therefore, effective treatment approaches.
• Molecular docking studies and structure–activity models can play an important role in identifying potent molecules for further exploration in *in vitro*, *in vivo,* and clinical studies.
• The use of disruptive technologies, including machine and deep learning and artificial intelligence, can accelerate drug discovery using comprehensive risk and efficacy analysis.
• Network pharmacology can reveal the evidence behind the potential efficacy of complementary medicine in CRPC.

The present study attempts to provide an overview of a rapidly expanding topic. It provides insights and guidance for future studies that will examine specific elements mentioned in this analysis, such as molecular docking or structure–activity modeling. Relevant future studies may benefit from a systematic review and a metanalysis methodology assessing both the available quantitative and qualitative evidence. Disruptive technology is expected to play an important role in future research. Artificial intelligence models based on deep machine learning can broadly analyze several natural compounds and their interactions. Quantum computing can act as an accelerator in such studies enabling the screening of big databases.

The adoption of a translational approach is a critical step toward clinical practice and application. Computational analysis of the effect of natural compounds on CRPC in combination with specific biomarkers extracted from the patients' histopathological specimens can lead to the identification of compounds that may be beneficial. The clinical outcomes of the patients receiving these compounds and their regular treatment can be compared with those of the control groups. Nonetheless, such an approach would be possible only using natural compounds that have been approved for clinical use by the respective health authorities. The use of a broader number of natural compounds might be possible in end-stage CRPC under compassionate authorization. However, such studies might reduce the efficacy of natural products due to the poor prognosis of the patients. In other words, it should be understood that the results of these studies do not necessarily reflect the efficacy of natural compounds administered at earlier stages of the disease.

Finally, yet importantly, it is important to integrate social and health economics factors in this research. Cultural factors have been shown to affect patients' attitudes toward several anticancer drugs; moreover, the attitudes toward natural compounds need to be assessed. People of different nationalities may be more open to receiving phytochemicals that they are already familiar with because of their cultural background. On top of this, the integration of phytochemicals as supplements in anticancer treatment may be evaluated in terms of cost-effectiveness. This will determine whether insurance systems and policymakers are willing to make such treatments widely available to the public. In the long run, patients, physicians, and regulatory authorities will accept the use of natural compounds for managing CRPC, which will result in conducting more large-scale studies and collecting better evidence and data.

As advanced-stage PCas are prone to the extensive point mutations that lead to drug resistance, single-targeted and specific drugs are no longer beneficial and cannot go further in clinical trials. In contrast, many natural products, such as resveratrol, soy isoflavone, lycopene, quercetin, gossypol, EGCG, and curcumin, progressed to clinical studies because of their multitarget anticancer potential.

Moreover, formulation advancements and the discovery of potent natural products play a crucial role in overcoming the limitations concerning the poor pharmacokinetics and bioavailability of natural products. Nanocurcumin-based clinical studies are a small step toward addressing the solubility and bioavailability related issues.

Utilizing all means available to integrate the use of natural compounds sources into clinical practices can be extremely beneficial in the management of CRPC in the future.
